# Joint transcriptomic and metabolomic analysis provides new insights into drought resistance in watermelon (*Citrullus lanatus*)

**DOI:** 10.3389/fpls.2024.1364631

**Published:** 2024-05-03

**Authors:** Sheng Chen, Kaiqin Zhong, Yongyu Li, Changhui Bai, Zhuzheng Xue, Yufen Wu

**Affiliations:** ^1^ Crops Research Institute, Fujian Academy of Agricultural Sciences, Fuzhou, China; ^2^ Fuzhou Institute of Vegetable Science, Fuzhou, China; ^3^ College of Horticulture, Fujian Agriculture and Forestry University, Fuzhou, China

**Keywords:** watermelon, drought, RNA-Seq, metabolomic, WGCNA

## Abstract

**Introduction:**

Watermelon is an annual vine of the family *Cucurbitaceae*. Watermelon plants produce a fruit that people love and have important nutritional and economic value. With global warming and deterioration of the ecological environment, abiotic stresses, including drought, have become important factors that impact the yield and quality of watermelon plants. Previous research on watermelon drought resistance has included analyzing homologous genes based on known drought-responsive genes and pathways in other species.

**Methods:**

However, identifying key pathways and genes involved in watermelon drought resistance through high-throughput omics methods is particularly important. In this study, RNA-seq and metabolomic analysis were performed on watermelon plants at five time points (0 h, 1 h, 6 h, 12 h and 24 h) before and after drought stress.

**Results:**

Transcriptomic analysis revealed 7829 differentially expressed genes (DEGs) at the five time points. The DEGs were grouped into five clusters using the k-means clustering algorithm. The functional category for each cluster was annotated based on the Kyoto Encyclopedia of Genes and Genomes (KEGG) database; different clusters were associated with different time points after stress. A total of 949 metabolites were divided into 10 categories, with lipids and lipid-like molecules accounting for the most metabolites. Differential expression analysis revealed 22 differentially regulated metabolites (DRMs) among the five time points. Through joint analysis of RNA-seq and metabolome data, the 6-h period was identified as the critical period for watermelon drought resistance, and the starch and sucrose metabolism, plant hormone signal transduction and photosynthesis pathways were identified as important regulatory pathways involved in watermelon drought resistance. In addition, 15 candidate genes associated with watermelon drought resistance were identified through joint RNA-seq and metabolome analysis combined with weighted correlation network analysis (WGCNA). Four of these genes encode transcription factors, including bHLH (*Cla97C03G068160*), MYB (*Cla97C01G002440*), HSP (*Cla97C02G033390*) and GRF (*Cla97C02G042620*), one key gene in the ABA pathway, *SnRK2-4* (*Cla97C10G186750*), and the *GP-2* gene (*Cla97C05G105810*), which is involved in the starch and sucrose metabolism pathway.

**Discussion:**

In summary, our study provides a theoretical basis for elucidating the molecular mechanisms underlying drought resistance in watermelon plants and provides new genetic resources for the study of drought resistance in this crop.

## Introduction

Watermelon (*Citrullus lanatus*) is an annual vine in the family *Cucurbitaceae*. Watermelon plant is a popular fruit crop with important nutritional and economic value. As one of the main summertime fruits and vegetables with sufficient supply and strong market demand, watermelon has a substantial agricultural output value (Deng et al., 2022). Moreover, watermelon fruits also contain nutrients such as vitamin C, vitamin A, and potassium, which promote human health and provide additional water and a variety of vitamins needed by the body (Volino-Souza et al., 2022). Watermelon plants produce low-calorie and high-fiber fruits that help control weight, promote digestive health, and contain natural antioxidants (Naz et al., 2014). With global warming and increasing shortages of water resources, drought has become one of the main abiotic stresses that impacts the normal growth of plants and substantially reduces the yield and quality of watermelon plants; thus, drought is one of the main problems faced during watermelon cultivation (Malambane et al., 2023). Therefore, studying the mechanism underlying watermelon drought resistance and identifying key drought resistance genes are highly important for ensuring the sustainable development of the watermelon industry (Malambane et al., 2023).

After drought stress, plants undergo a series of morphological and physiological changes to adapt to changes in external conditions (Godwin and Farrona, 2020). The plant leaf is an important organ that produces organic nutrients, and it is involved in photosynthesis, respiration and transpiration (Hikosaka, 2005). Under drought conditions, water in the soil is severely lacking, which results in plant leaves being prone to withering, yellowing and even dying, and plants become unable to maintain normal physiological activities (Chen et al., 2021; Guo et al., 2023). Leaf water loss strongly affects photosynthesis and reduces the capacity of plants to synthesize nutrients (Sun et al., 2022). Drought can also arrest watermelon fruit development, which affects fruit production and ripening and thereby reduces yield (Morales et al., 2023). When drought occurs, the size of the fruit may decrease, or fruit growth may be stunted, which can affect the taste, sweetness, and nutritional value of the fruit, reduce sales value and market competitiveness, and lead to economic losses for farmers and producers (Morales et al., 2023). Previous research has shown that after 8 days (d) of drought stress during the watermelon seedling stage, the relative water content (RWC) of dry watermelon cultivars Y34 (drought-sensitive material) and M20 (tolerant material) significantly decreased by 38.78% and 13.68%, respectively, but the MDA content increased by 83.05% and 40.89%, respectively. The total soluble sugar and proline contents of watermelon leaves after drought stress treatment for 12 days were 24.60% and 168.48% greater, respectively, than those before treatment (Li et al., 2019). Several studies have shown that the leaf water content does not decrease after 3 days of drought stress. This finding indicates that some solutes may accumulate to reduce the water potential, but the accumulation is not significant because the turgor pressure decreases with decreasing leaf water content. Mo et al. reported that eight days after the beginning of drought stress, the water content of the fourth leaf remained at 81% (2016). After drought stress, plants synthesize large amounts of abscisic acid (ABA). ABA signals close plant leaf stomata to reduce water evaporation, but this also prevents carbon dioxide from entering mesophyll cells and limits photosynthesis (Lim et al., 2015). Drought stress can change the chlorophyll structure, degrade photosynthetic pigments, destroy the photosynthetic electron system, and decrease the activity of photosynthesis-related enzymes, such as RuBP carboxylase and pyruvate kinase; additionally, the structure and function of photosystem II can be damaged, resulting in a decrease in the activity of photosynthesis-related enzymes (Helm et al., 2020). Oxygen accumulation damages the membrane system, which in turn disrupts photosynthesis and reduces production (Yang et al., 2019).

With the rapid development of sequencing technology and bioinformatics, transcriptomics, proteomics, metabolomics, ionomics and other technologies have been applied to the study of drought resistance mechanisms in plants (Singh et al., 2016). Multiomics analysis helps to comprehensively reveal the mechanisms of resistance in plants (Zargar et al., 2022). Metabolomics is an emerging omics technology that involves qualitative or quantitative analysis of metabolite contents in plants. In contrast to transcriptomics and proteomics, metabolomics is a direct indicator of plant phenotype (Ghatak et al., 2018). This technique provides more intuitive information on plant changes and can be a powerful tool for the study of plant responses to stress (Ghatak et al., 2018). The joint analysis of transcriptomics and metabolomics results can unify the information obtained at the gene and metabolite levels and be used to elucidate the overall molecular mechanisms underlying plant responses to abiotic stresses (Huan et al., 2022; Tiedge et al., 2022). Combined analysis of the transcriptome and metabolome of *Sophora chinensis* revealed that the biosynthesis of flavonoid metabolites is important in response to drought stress (Huang et al., 2023). Analysis of the transcriptome, proteome and metabolites of *Craterostigma plantagineum* leaves during dehydration and rehydration cycles revealed that the photosynthesis and photorespiratory pathways are important pathways in the drought resistance of *C. plantagineum*, and the origins of these pathways have been proposed (Xu et al., 2021). Multiomics analysis revealed that the 2-oxocarboxylate metabolism and isoflavone biosynthesis pathways were the core pathways involved in mepiquine-mediated regulation of the drought response in soybean, and candidate genes involved in these processes were also identified (Wang et al., 2023b). WGCNA combined with metabolomic and transcriptomic analyses has indicated that the sucrose metabolism pathway genes *AMY3* and *CWINV1* are responsible for the relatively high sucrose content in the roots of *Schisandra chinensis*, which in turn promoted the development of the root system and increased drought resistance (Yin et al., 2023). In another study, the transcriptome and metabolome of *Cyclocarya paliurus* under different PEG concentrations were analyzed, and WGCNA revealed that 3 key modules, 8 structural genes and 14 regulatory transcription factors regulate sugar metabolism under drought stress (Li et al., 2023a). By combining transcriptomics and metabolomics, researchers showed that *OsCIPK17* positively regulates drought resistance in rice and is involved in the accumulation of citrate in the TCA cycle (Lu et al., 2023). Transcriptome and metabolome analyses of *Illicium difengpi* under drought stress and water replenishment revealed that there were more genes with upregulated expression than genes with downregulated expression, and water replenishment treatment induced the stable expression of 65.25% of genes (Zhang et al., 2024).

The completion of watermelon genome sequencing has facilitated the study of watermelon genomics, functional omics, and the regulation of various biological processes under environmental stress using transcriptome technology (Deng et al., 2022). For example, RNA-seq and metabolomic analyses revealed the regulatory network and mechanisms underlying the production of sugars and organic acids in watermelon 10, 18, 26, and 34 days after pollination (Umer et al., 2020). The overexpression of watermelon *ClMTB* has been found to increase the drought resistance of transgenic tobacco plants, and further analysis revealed that *ClMTB* improved drought resistance by enhancing the reactive oxygen species (ROS) scavenging system and alleviating photosynthesis inhibition under drought conditions (He et al., 2021b). Through the identification of the HSP70 gene family and expression analysis, researchers found that HSP70 is induced when watermelon is subjected to drought stress (Wang et al., 2023a). Through a genome-wide analysis of the watermelon plant AT protein and zinc-binding protein (PLATZ) family, researchers demonstrated that *ClPLATZ8* and *ClPLATZ11* can improve the drought resistance of transgenic Arabidopsis (Qi et al., 2023). Based on the expression analysis of 16 O-methyltransferase (*ClOMT*) genes in watermelon, the expression of *ClOMT1* can be induced by stress. The secondary protein is located in the cytoplasm, and its overexpression enhances the tolerance of transgenic Arabidopsis to abiotic stress (Chang et al., 2021). In the early years of this research field, there was only one report each on the transcriptome and proteome of watermelon under drought resistance, but this was a relatively long time ago. Currently, the identification of watermelon drought-related genes and related research are still at the stage of gene family analysis, which involves finding homologous genes based on known drought-responsive genes and pathways in other species (Kawasaki et al., 2000; Yang et al., 2016). However, identifying key pathways and genes involved in watermelon drought resistance through high-throughput omics methods is particularly important. The seedling stage is the stage of plant growth and development at which the plant is most sensitive to drought stress. Studying the molecular mechanisms involved in the response of watermelon plants to drought stress at the seedling stage and identifying drought resistance genes are key for improving watermelon yield. Therefore, in the present study, transcriptome and metabolome sequencing technologies were used to study watermelon plants at five time points before and after drought stress. K-means cluster analysis and Kyoto Encyclopedia of Genes and Genomes (KEGG) enrichment analysis were performed on the differentially expressed genes (DEGs) and metabolites, regulatory pattern analysis was performed on the metabolites and genes in key pathways, and coexpression analysis was performed to identify the key genes involved in drought resistance in watermelon. This study provides new insights into watermelon drought resistance and reveals key regulatory pathways and candidate genes involved in watermelon drought resistance.

## Materials and methods

### Plant materials and drought treatment

In this study, the watermelon cultivar black jade (cultivated by the Institute of Agro-Biological Resources of Fujian Academy of Agricultural Sciences, Chinese Variety Registration Number: GPD Watermelon (2021) 350012) was chosen for research. The varietal characteristics were as follows: hybrid variety, medium-sized watermelon, fruit development period of 26 days, and full growth period of 80 days. The first female flower position is 8, with an interval of 4 between the appearance of female flowers. The fruit is oval-shaped, with waxy skin, a single fruit weight of 4.5 kilograms, a red flesh color, a fruit skin thickness of 1 centimeter, a green base color of the skin, and hidden skin stripes. The soluble solids content in the center is 11.2%, while that in the edge is 9.1%. The fruit skin is hard, has fine, crisp and juicy flesh, has good toughness, and is suitable for transportation. The plant is susceptible to wilt disease and shows strong drought resistance. The yield per mu in the first growth cycle was 2150.7 kilograms, an increase of 11.03% compared to that of the control, black beauty. The yield per mu in the second growth cycle was 2200.9 kilograms, an 11.20% increase compared to that of the control, black beauty. Seeds with good maturity and plump and uniform grains were selected, soaked in warm water at 55°C for 4 h, and subsequently placed in the dark at 30°C for accelerated germination. Seedlings were grown in a greenhouse and watered with 1/2 Hoagland nutrient solution every day. The day and night temperatures of the greenhouse were 28/20°C, the air humidity was 65%, the light conditions were 16 hours of light and 8 hours of darkness, and the light source was fluorescent lamps. After the second true leaf of the plants unfolded, plants with consistent growth were selected, the substrate adhering to the roots was carefully removed, and the plants were subsequently placed in a hydroponic box for hydroponic cultivation. The hydroponic method was performed as follows: a 30 L plastic box was used as a hydroponic box, the culture nutrient solution was changed once a week (Hoagland nutrient solution), foam boards were cut to match the length and width of the incubator, the incubators were covered with foam boards, and spaces were evenly made on the foam boards. When watermelon plants had 5-6 true leaves, the nutrient solution in the hydroponic tanks was replaced with 20% PEG6000. The net photosynthetic rate (Pn), transpiration rate (Tr), intercellular carbon dioxide (Ci) and stomatal conductance (Gs) were measured with an LI-6400 portable photosynthetic instrument after 20% PEG6000 treatment for 0 h, 1 h, 6 h, 12 h and 24 h (Lawrence et al., 2019). Moreover, 11 replicates of each sample (3 for RNA-seq, 5 for metabolome sequencing, and 3 for qRT−PCR) were collected, wrapped in aluminum foil, quickly placed in liquid nitrogen and subsequently stored at -80°C.

### RNA-seq library construction, sequencing, and analysis

A polysaccharide and polyphenol plant total RNA extraction kit was used to extract RNA (each sample was approximately 100 mg). The extraction process was carried out according to the manufacturer’s instructions. After RNA extraction, a Nanodrop was used to determine whether the purity (OD260/280), concentration, and nucleic acid absorbance peak of the RNA were normal. An Agilent 2100 was used to accurately determine the integrity of the RNA. The indicators included the RIN, 28S/18S ratio, whether the spectral baseline was increased and the presence of the 5S peak. The concentration of RNA in all the samples was ≥600 ng/μl, and the total amount of RNA was ≥2 μg. The samples that met the criteria were shipped to Beijing Biomics Biotech Co., Ltd. (Beijing, China) for sequencing. Oligo (dT) magnetic beads were used to enrich eukaryotic mRNA through the binding of AT complementary pairs to the poly(A) tail of the mRNA. Then, fragmentation buffer was added to fragment the mRNA into short fragments. The mRNA was used as a template to synthesize first-strand cDNA with random hexamers, followed by the addition of buffer, dNTPs, and DNA polymerase I to synthesize second-strand cDNA, which was subsequently synthesized by AMPure. Double-stranded cDNA was purified using XP beads. The purified double-stranded cDNA was end-repaired and A-tailed, and sequencing adapters were ligated. The fragments were subsequently selected using AMPure XP beads, after which PCR enrichment was performed to obtain the final cDNA library. An Agilent 2100 was used to determine the size of the insert in the library. The Q-PCR method was used to accurately quantify the effective concentration of the library (effective concentration of the library>2 nM) to complete the library check. The constructed libraries were sequenced on the Illumina HiSeq 2500 sequencing platform. After obtaining the raw sequences, fastp software (version 0.23.4) was used to remove adapter sequences and filter out low-quality N sequences with a proportion greater than 5%, etc. to obtain clean reads for use in subsequent analyses (Chen et al., 2018). The clean reads were aligned to the watermelon reference genome (http://cucurbitgenomics.org/v2/organism/16) using HISAT2 software (Kim et al., 2019). The fragments per kilobase of exon model per million mapped fragments (FPKM) were used to determine the expression level. The P value and fold change were calculated with DESeq2 software, with a P value ≤ 0.05 and |log2fold change|>1 serving as the screening criteria for identifying DEGs according to differential expression (Liu et al., 2021). The DEGs were analyzed based on the KEGG database (http://www.genome.jp/kegg/) for the annotation of gene function.

### Metabolite extraction and detection

After the tissue samples were stored at -80°C, placed at -20°C for 30 min and subsequently placed in a refrigerator at 4°C for thawing, 25 mg of sample was weighed and added to precooled methanol/acetonitrile/water solution (2:2:1). After vortexing, the mixture was sonicated at low temperature for 30 min, held at -20°C for 10 min, and centrifuged at 14,000 × g for 20 min at 4°C. The supernatant was collected and dried under vacuum. During mass spectrometry analysis, the solution was reconstituted by adding 100 μL of acetonitrile aqueous solution (acetonitrile:water = 1:1) followed by vortexing. The mixture was centrifuged at 14,000 × g for 15 min at 4°C, after which the supernatant was collected for sample analysis. The samples were separated on an Agilent 1290 Infinity LC ultrahigh-performance liquid chromatography (UHPLC) C-18 column (column temperature, 40°C; flow rate, 0.4 mL/min; injection volume, 2 μL; mobile phase composition, A: water + 25 mM acetic acid; ammonium + 0.5% formic acid; and B: methanol). The gradient elution program was as follows: 0.5 min, 5% B; 0.5–10 min, B was changed linearly from 5% to 100%; 10.0–12.0 min, B was maintained at 100%; B was changed linearly from 100% to 5% at 12.0–12.1 min; B was maintained at 5% at 12.1–16 min; and the samples were placed in an autosampler at 4°C during the entire analysis. The primary and secondary spectra of the samples were collected using an AB Triple TOF 6600 mass spectrometer. The raw data in the Wiff format were converted to the.mzXML format by ProteoWizard, and MS-DAIL software was subsequently used for peak alignment, retention time correction and peak area extraction. Using the data extracted by MS-DAIL, the first metabolite structure was identified, the data were preprocessed, the experimental data quality was evaluated, and data analysis steps were performed (Tsugawa et al., 2015).

### Metabolomic analysis

In this study, the metabolomics database of the New Life Plants of the Chinese Academy of Sciences was used [in-house database (Shanghai Applied Protein Technology)]. The structures of the metabolites in the biological samples were identified by matching the molecular mass (molecular mass error within <10 ppm), second-order fragmentation spectrum, and retention time of the metabolites in the database, and the identification results were analyzed, checked and confirmed. Based on the metabolite content data matrix, principal component analysis (PCA) was performed on each sample using the R 4.2.3 language PCAtools software package (R package version 2.14). The Human Metabolome Database (HMDB, Version 5.0) and the KEGG database (https://www.genome.jp/kegg/) were used to classify the metabolites and functionally annotate the pathways to determine the main biochemical metabolic pathways and signal transduction pathways associated with the metabolites (Kanehisa and Goto, 2000). Partial least squares regression was used to construct a model of the relationship between the expression levels of metabolites and sample categories to predict sample categories (Pi et al., 2023). The fold change in the expression of each metabolite in each comparison group was calculated, and a significance test was performed on the expression level of each metabolite in each comparison group using Student’s t test to obtain the q value (corrected p value for the t test). A |log2fold change|>1 and q value < 0.05 were used as the standards for identifying differentially abundant metabolites.

### WGCNA

The WGCNA package of the R package was used for coexpression analysis of DEG expression profiles via the dynamic branch and cut method (Langfelder and Horvath, 2008). The weighting factor β should satisfy a correlation coefficient close to 0.8. In this study, β=4 was chosen as the weighting factor. The network was constructed using the automatic network construction function “Blockwise Modules” to obtain gene coexpression modules. Each module contained different numbers of genes. The modules with minModuleSize = 30 and merge cut height = 0.25 were used as standards to merge modules with a similarity of 0.75. The correlation coefficients between the characteristic vector ME (module eigen) of the module and Pn, Tr, Ci, Gs, ABA, SA and JA were calculated. Cytoscape (version 3.10.1) software was used for visualization of the coexpression network (Shannon et al., 2003).

### qRT−PCR

Total RNA was extracted using an RNAprep Pure Polysaccharide and Polyphenol Plant Total RNA Extraction Kit (Tiangen, China). The concentration of each RNA sample was determined using a NanoDrop 2000 spectrophotometer (Thermo Fisher Scientific, Waltham, MA, USA). cDNA was obtained by reverse transcription of RNA using an M-MLV RTase cDNA Synthesis Kit (TaKaRa, Japan). qRT−PCR analysis was performed using a Roche LC480 instrument (Roche Diagnostics GmbH, Mannheim, Germany) and SYBR Green (Takara Bio, Inc.). The volume of the reaction mixture was 20 μL. The reaction program was set as follows: predenaturation at 95°C for 30 s; denaturation at 95°C for 5 s; annealing at 60°C for 5 s; and extension at 72°C for 30 s (35 cycles). The results were quantitatively analyzed by the 2^–ΔΔCt^ method. The Ct value of the internal reference gene was subtracted from the Ct value of the target gene to obtain the ΔCt. The mean ΔCt value of the control group (0 h) was subtracted from each ΔCt of the treatment group to obtain the ΔΔCt. The final expression level was determined based on the equation 2^–ΔΔCt^ (Livak and Schmittgen, 2001). The internal reference gene was ACTIN (*Cla97C02G026960*), and three biological replicates were performed for each procedure. All primers used in this study are listed in **Supplementary Table S1**.

### Statistical analysis

In this study, Excel 2010 was used for Pn, Tr, Ci, Gs and qRT−PCR data statistics, and the R ggplot2 software version was used for light and data visualization (Pn, Tr, Ci and Gs). The data before 0 h drought stress (CK) and after stress were tested using the ggpubr package to calculate the significance level. Transcriptome and metabolome heatmaps were drawn using the R pheatmap software package, and correlation analysis was performed using the corrplot software package. The correlation and p values for genes and metabolites were calculated using the R Hmisc software package, and the network diagram was visualized using Cytoscape (version 3.10.1) software (Shannon et al., 2003).

## Results

### Changes in light and other indicators in watermelon plants under drought stress

**Figure 1:**
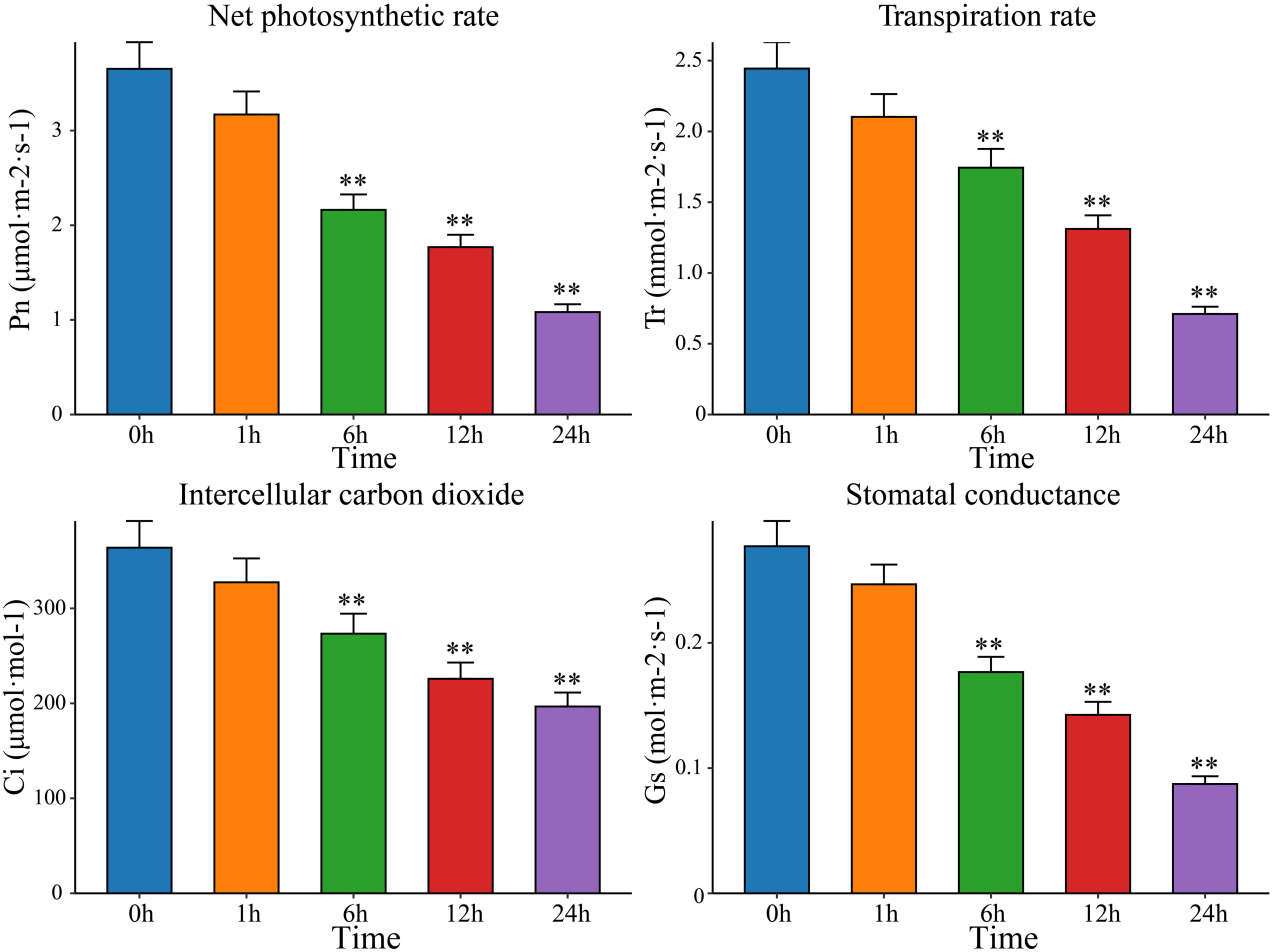
The net photosynthetic rate (Pn), transpiration rate (Tr), intercellular carbon dioxide (Ci) and stomatal conductance (Gs) in leaves at five time points before and after drought stress (0 h, 1 h, 6 h, 12 h and 24 h). The results are presented as the means ± SDs (n = 3, **P < 0.01).

After drought stress, plants undergo a series of morphological and physiological changes to adapt to external conditions. The openings of the leaf stomata decrease in size or even close, thus hindering the entry of CO_2_ into the leaves, affecting the utilization of CO_2_ in the carboxylation center, and reducing the rate of photosynthesis. Therefore, measuring photosynthetic indicators and analyzing the response of plants to drought stress can help elucidate the process and mechanism underlying the impact of drought stress on plant photosynthesis; this information can be used to scientifically formulate strategies for breeding drought -resistant plants. Therefore, the Pn, Tr, Ci and Gs of watermelon leaves were measured after exposure to drought stress for 0 h, 1 h, 6 h, 12 h, and 24 h. Compared with the Pn, Tr, Ci, and Gs in watermelon leaves before treatment, the Pn, Tr, Ci, and Gs in leaves exposed to drought stress decreased significantly with increasing drought stress time, reaching a minimum at 24 h (**Figure 1**). Pn and Tr decreased the most, with Pn at 29.64% of the pretreatment level and Tr at 29.07%. Ci and Gs decreased by 48.39% and 54.04%, respectively, of the pretreatment levels. This study showed that plants adapt to drought by changing their stomatal density, which affects photosynthesis through changes in intercellular CO_2_ concentrations. Watermelon mainly reduces Pn levels by regulating Gs and Tr and closing stomata to reduce CO_2_ intake, thereby mitigating the damage to its photosynthetic system caused by drought stress. To further identify key genes and metabolites involved in watermelon drought stress, RNA-seq and metabolomics sequencing were performed on leaves at five time points (0 h, 1 h, 6 h, 12 h and 24 h) before and after drought stress.

### RNA-seq analysis

**Figure 2:**
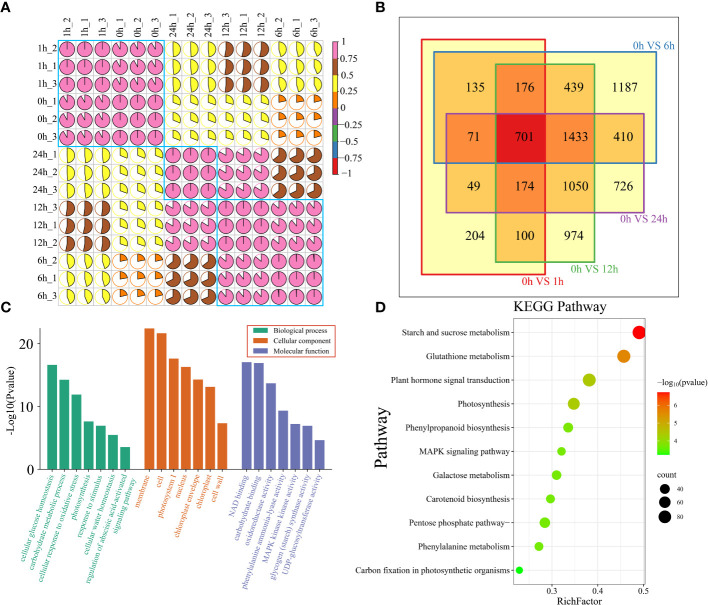
**(A)** Correlation and cluster analysis of the 15 RNA-seq samples before and after drought stress in watermelon. **(B)** Venn diagram of DEGs at different time points and 0 h after drought treatment. **(C)** All DEG GO features are annotated and categorized. There are three levels of GO enrichment, and different levels are distinguished by different colors. The level of significance is expressed by the height of the column. The higher the column is, the smaller the P value and the more significant the enrichment is. **(D)** All DEG KEGG pathway annotations. The color represents the P value. The larger the -log10(P value) and the smaller the P value are, the more significant the pathway is.

A total of 102.53 Gb of raw RNA-seq data were obtained from 15 watermelon samples collected at five time points (0 h, 1 h, 6 h, 12 h and 24 h) before and after drought stress. After filtering, a total of 99.99 Gb of clean data were obtained. The amount of clean data from each sample exceeded 5.81 Gb, the Q30 base percentage exceeded 90.67%, the GC content exceeded 41.71%, and the alignment rate with the reference genome exceeded 91.96% (**Supplementary Table S2**). The correlation coefficients between the same sample and different biological replicates were all greater than 0.95. The PCA results showed that the biological replicate s were clustered together, indicating that the transcriptome data were reliable and repeatable (**Figure 2A**; **Supplementary Figure S1**). To analyze the transcriptome dynamics of watermelon drought stress, cluster analysis and principal component analysis (PCA) were performed on RNA-seq data from 15 samples collected at 5 time points (0 h, 1 h, 6 h, 12 h and 24 h). The RNA-seq data was divided into 3 groups. The samples at the earliest time points (0 h and 1 h) formed the first group, which represented the early stage of stress. The samples at 6 h and 12 h formed the second group, representing the middle stage of stress, during which the most DEGs were produced. The 24 h sample belongs to the third group, corresponding to the later stage of stress. The clustering of the earliest stress time points (0 h and 1 h) and 24 h was closer, indicating that their transcriptional regulation patterns were highly similar.

To study the regulatory mechanisms of transcription of watermelon plants at different stages of drought stress, DEGs at each time point during drought stress were identified (**Figure 2B**). There were 1610 DEGs at 1 h compared to that at 0 h. There were 4552 DEGs at 6 h, 5047 DEGs at 12 h, 4614 DEGs at 24 h, and a total of 701 shared DEGs at the four time points (**Figure 2B**). To elucidate the functions of all 7829 DEGs, DEG enrichment was determined by GO term and KEGG pathway analyses (**Supplementary Table S3**). The results showed that the most significantly enriched terms in the cellular component subclass were related to the membrane and cell. The most prominent terms in the molecular function subcategory were involved in NAD and carbohydrate binding, while those in the biological process category were involved in cellular glucose homeostasis and carbohydrate metabolism (**Figure 2C**). KEGG enrichment analysis of all DEGs revealed that the DEGs were involved mainly in starch and sucrose metabolism, glutathione metabolism, plant hormone signal transduction, and photosynthesis (**Figure 2D**).

**Figure 3:**
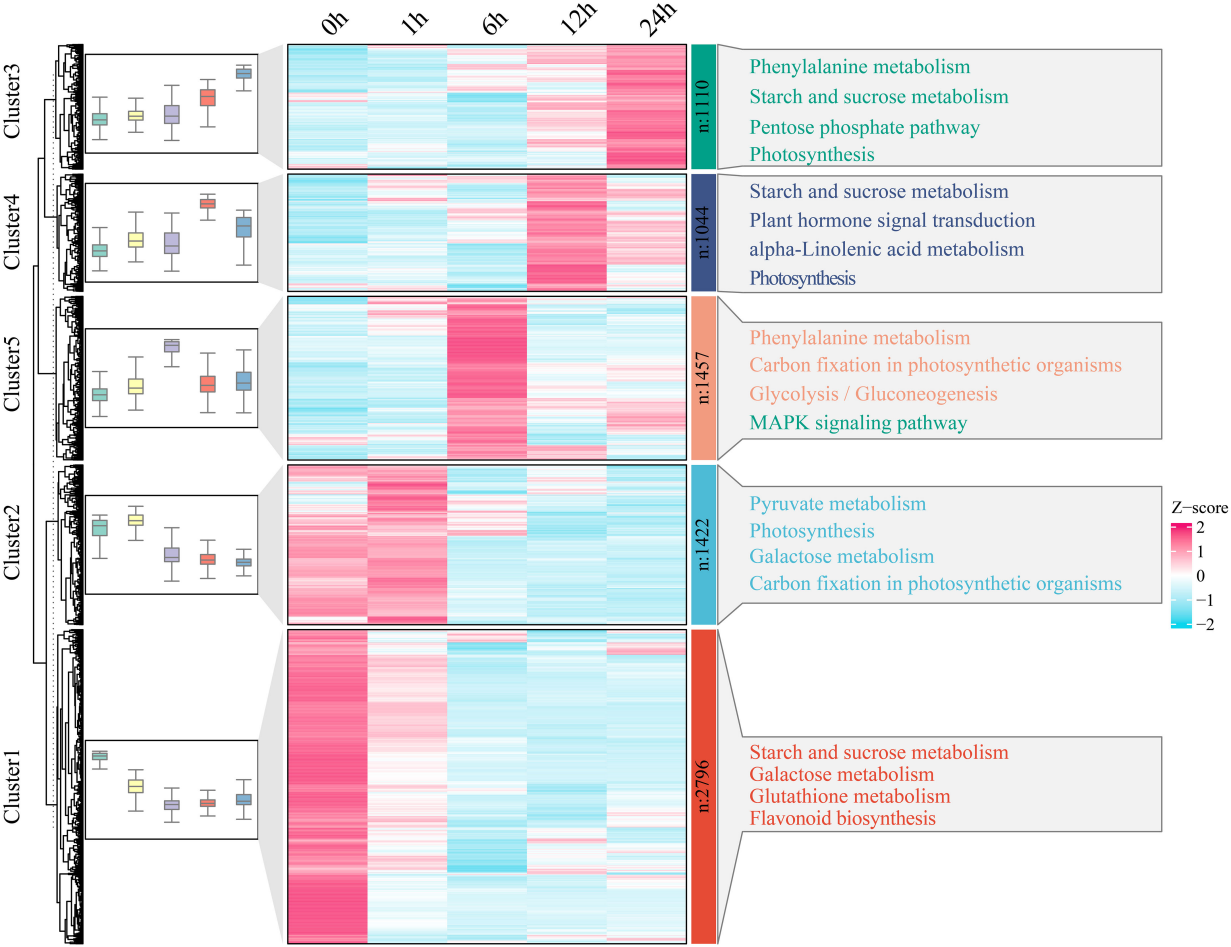
With respect to clusters of DEGs and KEGG pathway enrichment analysis, the expression levels of DEGs are depicted as heatmaps, and the data were z score normalized during analysis; the larger the value was, the greater the expression level, with the highest being 2. The smaller the value is, the lower the expression level, with the lowest being -2.

To further investigate the functional transformation among genes associated with watermelon drought stress, the 7829 genes that were differentially expressed in all periods were clustered into five clusters using the k-means clustering algorithm, and then KEGG analysis was performed to annotate the functional category of each cluster (**Figure 3**). Cluster 1 was specifically and highly expressed at 0 h and was enriched mainly in the starch and sucrose metabolism, galactose metabolism, glutathione metabolism and flavonoid biosynthesis pathways. Cluster 2 was highly expressed at 0 h and 1 h and was mainly enriched in pyruvate metabolism, photosynthesis, galactose metabolism and carbon fixation in photosynthetic organism pathways. Cluster 3 was highly expressed at 6 h and was mainly enriched in terms related to phenylalanine metabolism, carbon fixation in photosynthetic organisms, glycolysis and gluconeogenesis, and the mitogen-activated protein kinase (MAPK) signaling pathway. Cluster 4 was highly expressed at 12 h and was mainly enriched in the starch and sucrose metabolism, plant hormone signal transduction, alpha-linolenic acid metabolism and photosynthesis pathways. Cluster 4 was highly expressed at 24 h, and the main annotations involved phenylalanine metabolism, starch and sucrose metabolism, the pentose phosphate pathway and photosynthesis pathways.

### Metabolomic analysis

**Figure 4:**
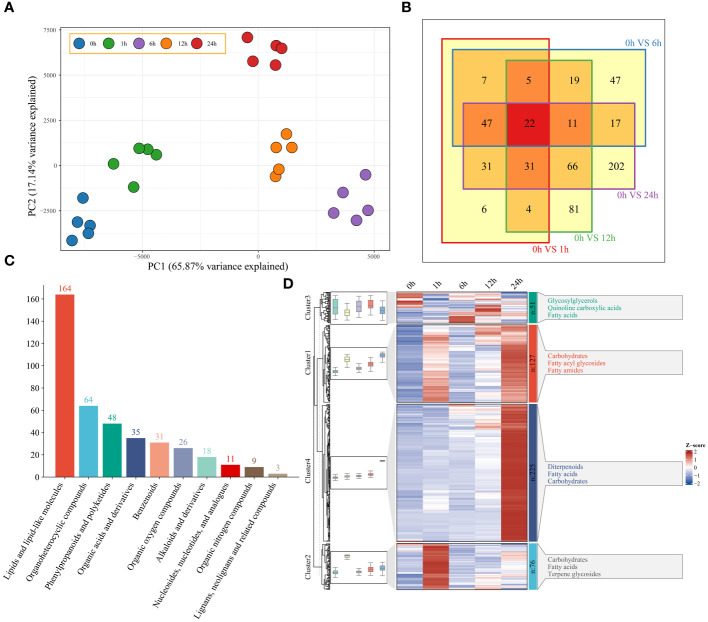
**(A)** PCA of 25 metabolome samples of watermelon before and after drought stress. **(B)** The differences in the metabolome at different time points and before stress were analyzed via a Venn diagram. **(C)** Histogram of all different metabolic classifications. **(D)** Clustering and metabolite classification of all DRMs. The expression levels of metabolites are depicted in heatmaps, and the data were z score normalized during analysis; the larger the value, the greater the expression level, with the highest being 2. The smaller the value, the lower the expression level, with the lowest being -2.

Based on the 949 metabolites obtained by UPLC−MS, PCA revealed the segregation of 25 watermelon samples at five time points (0 h, 1 h, 6 h, 12 h and 24 h) before and after drought stress (**Figure 4A**). To understand the classification and functional properties of different metabolites, the identified metabolites were annotated. The annotated metabolites were divided into 10 main categories. There were 303 metabolites related to lipids and lipid-like molecules; 103 metabolites related to organoheterocyclic compounds; 97 metabolites related to phenylpropanoids and polyketides; and 14 metabolites related to lignans, neolignans and related compounds (**Supplementary Figure S2**). To study the changes in metabolites in watermelon at different stages of drought stress, a total of 479 differentially regulated metabolites (DRMs) were identified; compared to those at 0 h, there were 154 DRMs at 1 h, 176 DRMs at 6 h, 239 DRMs at 12 h, 428 DRMs at 24 h, and 22 DRMs at all four time points (**Figure 4B**; **Supplementary Table S4**). Lipids, lipid-like molecules and organoheterocyclic compounds exhibited the greatest differences (164 and 64, respectively) (**Figure 4C**). The changes in DRM content showed that these metabolites were affected by drought stress, and they were grouped into four clusters using the k-means clustering algorithm (**Figure 4D**). Cluster 1 had the highest levels at 6 h and 24 h and included carbohydrates, fatty acyl glycolosides and fatty amides. At 6 h, Cluster 2 exhibited the greatest increase in carbohydrate, fatty acid and terpene glycoside contents. Cluster 3 had the highest content at 0 h, 6 h and 12 h and included glycosylglycerols, quinoline carboxylic acids and fatty acids. Cluster 4 had the highest content of diterpenoids, fatty acids and carbohydrates at 24 h.

### Joint analysis of RNA-seq and metabolome data

**Figure 5:**
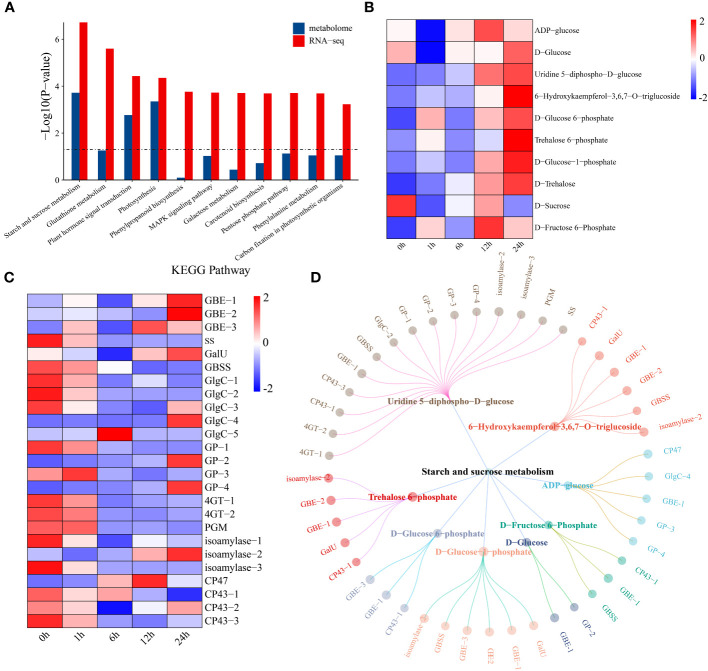
**(A)** KEGG enrichment analysis of DEGs and DRMs. **(B)** Changes in metabolite levels in the starch and sucrose metabolism pathways. The expression levels of metabolites were drawn as heatmaps, and the data were z score normalized during analysis. The larger the value, the greater the expression level, with the highest being 2. The smaller the value, the lower the expression level, with the lowest being -2. **(C)** Starch and sucrose metabolism pathway gene expression patterns. The expression levels of genes are depicted in heatmaps, and the data were z score normalized during analysis; the larger the value, the greater the expression level, with the highest being 2. The smaller the value, the lower the expression level, with the lowest being -2. **(D)** Correlation network diagram of starch and sucrose metabolism pathway genes and metabolites.

Through joint analysis of the transcriptome and metabolome, the relationships between 7829 DEGs and 479 DRMs under drought stress in watermelon were systematically explored. KEGG pathway enrichment analysis revealed that DEGs and DRMs were mainly enriched in 11 KEGG pathways, such as starch and sucrose metabolism, plant hormone signal transduction, and photosynthesis pathways (**Figure 5A**). First, changes in the levels of metabolites in the starch and sucrose metabolism pathways were analyzed. The levels of most metabolites increased with the prolongation of drought stress, while the D-sucrose content reached a minimum at 1 h of drought stress and then gradually increased, reaching a maximum at 12 h (**Figure 5B**). The expression levels of the glycogen biosynthesis pathway genes 1,4-alpha-glucan branching enzyme (GEB) and UTP-glucose-1-phosphate uridylyltransferase (GalU) both showed an upward trend, reaching a maximum level at 24 h, while the expression levels of starch synthase (ss), granule-bound starch synthase (GBSS) and glucose-1-phosphate adenylyltransferase (GlgC) decreased (**Figure 5C**). The expression of all genes in the glycogen degradation pathway (including glycogen phosphorylase (GP), 4-alpha-glucanotransferase (4GT) and phosphoglucomutase (PGM)) tended to increase. This result indicates that the increase in sugar content under drought conditions may be due to a decrease in glycogen degradation. A network of DEGs and DRMs in the starch and sucrose metabolism pathway was constructed using the screening criteria of PCC ≥ 0.75 and P < 0.05. The 15 genes that were most strongly regulated by uridine 5-diphospho-D-glucose and the other metabolites were regulated by 2-6 genes (**Figure 5D**). *GBE-1* was a key gene associated with eight starch and sucrose compounds.

**Figure 6:**
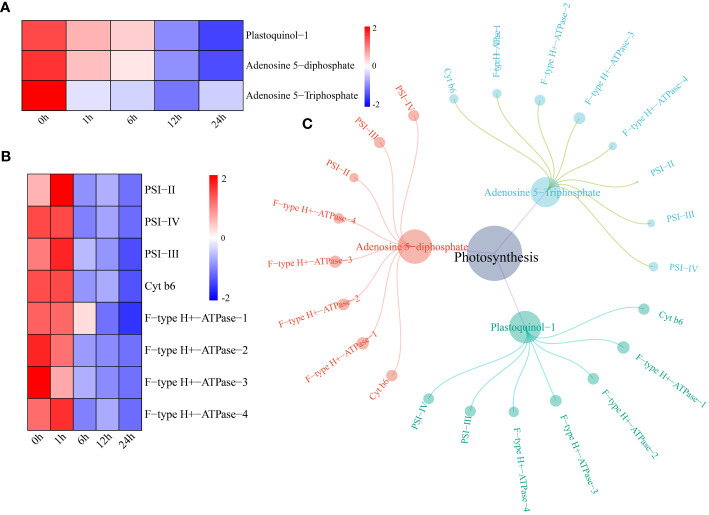
**(A)** Changes in metabolite content in the photosynthesis pathway. The expression levels of metabolites were drawn as heatmaps, and the data were z score normalized during analysis; the larger the value, the greater the expression level, with the highest being 2. The lower the value, the lower the expression level, with the lowest being -2. **(B)** For the photosynthesis pathway gene expression patterns, the expression levels of metabolites are depicted in heatmaps, and the data were z score normalized during analysis; the larger the value, the higher the expression level, with the highest being 2. The smaller the value, the lower the expression level, with the lowest being -2. **(C)** Correlation network diagram of photosynthesis pathway genes and metabolites.

The levels of two metabolites (plastoquinol-1 and adenosine 5-diphosphate) in the watermelon photosynthesis pathway began to decrease 12 h after drought stress, and the level of adenosine 5-triphosphate began to decrease after 1 h (**Figure 6A**). The expression levels of the photosynthesis pathway genes photosystem I subunit II (PSI-II), photosystem I subunit III (PSI-III), photosystem I subunit IV (PSI-IV), cytochrome b6 (Cyt b6) and F-type H+-transporting ATPase subunit a (F-type H+-ATPase) decreased after 6 h of drought stress (**Figure 6B**). A network of DEGs and DRMs in the photosynthesis pathway was constructed using the screening criteria of PCC ≥0.75 and P < 0.05. PSI-II regulated only adenosine 5-diphosphate and adenosine 5-triphosphate, and the remaining seven genes were shown to interact with three metabolites. These parameters were significantly correlated (**Figure 6C**).

**Figure 7:**
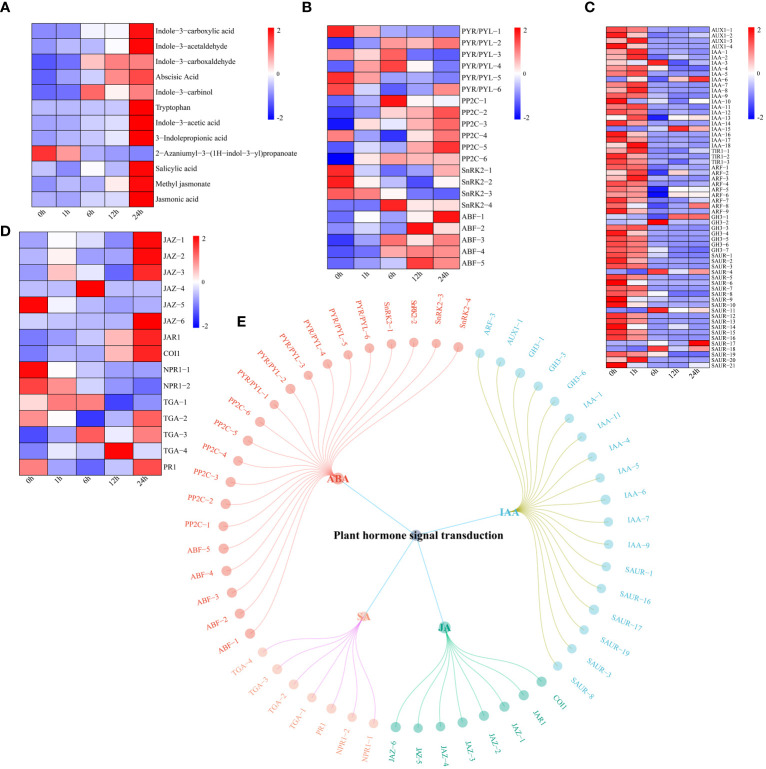
**(A)** Changes in the metabolite content of plant hormones (IAA, ABA, JA and SA), the expression levels of metabolites are depicted in heatmaps, and the data were z score normalized during analysis; the larger the value, the higher the expression level, with the highest being 2. The lower the value, the lower the expression level, with the lowest being -2. **(B)** Plant hormone ABA pathway gene expression patterns and the expression levels of genes are depicted as heatmaps, and the data were z score normalized during analysis; the larger the value, the higher the expression level, with the highest being 2. The lower the value, the lower the expression level, with the lowest being -2. **(C)** Plant hormone IAA pathway gene expression patterns and the expression levels of genes are depicted in heatmaps, and the data were z score normalized during analysis; the larger the value, the higher the expression level, with the highest being 2. The lower the value, the lower the expression level, with the lowest being -2. **(D)** Plant hormone JA and SA pathway gene expression patterns. The expression levels of genes are depicted in heatmaps, and the data were z score normalized during analysis; the larger the value, the higher the expression level, with the highest being 2. The lower the value, the lower the expression level, with the lowest being -2. **(E)** Correlation network diagram of plant hormone (IAA, ABA, JA and SA) pathway genes and metabolites.

UPLC−MS revealed significant changes in the levels of four hormones (ABA, IAA, JA and SA) in watermelon plants under drought stress (**Figure 7A**). The levels of all hormone-related metabolites increased except for those of 2-azaniumyl-3-(1H-indol-3-yl) propanoate. First, the expression levels of genes related to the ABA signaling pathway were analyzed. The expression levels of four pyrabactin-resistant/pyrabactin-resistant 1-L like (PYR/PYL) genes tended to decrease, while those of two genes tended to increase (**Figure 7B**). The expression levels of protein phosphatase 2C (PP2C) and ABA-responsive element (ABRE)-binding factor (ABF) both showed increasing trends. The expression of the three SNF1-related protein kinase 2 (SnRK2) genes decreased, while that of SnRK2-4 tended to increase, reaching a maximum at 6 h. These findings indicate that different genes in the ABA pathway respond to drought stress at different times. These genes may promote watermelon tolerance to drought stress. In contrast, the expression of most genes in the IAA pathway tended to decrease, which was contrary to the changes in IAA content (**Figure 7C**). Most genes in the JA pathway (except jasmonate ZIM-domain (JAZ)-5) had upregulated expression, and genes in the SA pathway, including nonexpressor of pathogenesis-related genes 1 (NPR1) and pathogenesis-related protein 1 (PR1) showed a downward trend, except for TGACG-binding (TGA)-3 and -4 which had upregulated expression (**Figure 7D**). Using the screening criteria of a PCC ≥ 0.75 and P < 0.05, a network of DEGs and DRMs in the plant hormone signal transduction pathway was constructed. Specifically, 21 genes regulated ABA, 18 genes regulated IAA, and eight genes regulated JA. SA was regulated by seven genes (**Figure 7E**).

### WGCNA

**Figure 8:**
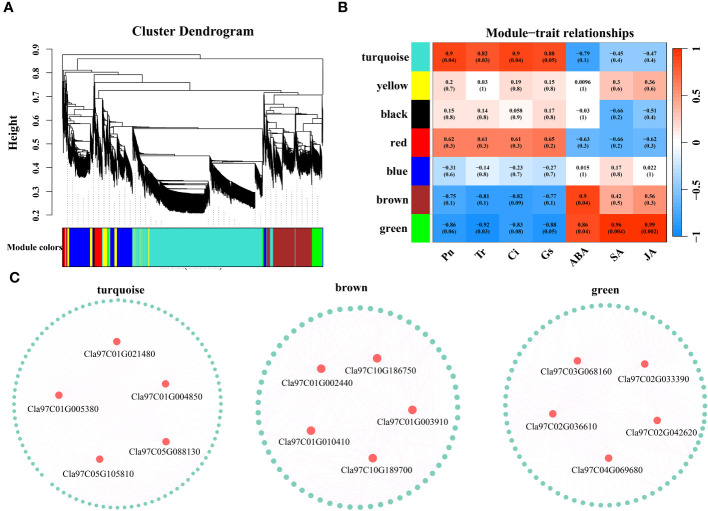
**(A)** Hierarchical clustering tree of genes based on coexpression network analysis. **(B)** Heatmap of the significant correlations between the modules and Pn, Tr, Ci, Gs, ABA, SA and JA. **(C)** Gene coexpression network within the turquoise, brown and green modules.

Based on the expression levels of the 7829 DEGs, β=4 was chosen for network construction, and the dynamic pruning tree method was used to merge modules with similar expression levels. A total of 7 coexpression modules were obtained, and different colors were used to represent different modules (**Figure 8A**). The calculated modules correlated with Pn, Tr, Ci, Gs, ABA, SA and JA (**Figure 8B**). The turquoise module was significantly correlated with Pn, Tr, Ci and Gs (r > 0.8, p < 0.05); the brown module was significantly correlated with ABA (r = 0.9, p < 0.05); and the green module was significantly correlated with ABA, SA and JA (r > 0.86, p < 0.05). For each module, the five genes with the highest connectivity were determined to be hub genes, and 15 hub genes were ultimately identified (**Figure 8C**). The 15 genes included one encoding a bHLH transcription factor (*Cla97C03G068160*), one encoding a MYB transcription factor (*Cla97C01G002440*), one encoding an HSP transcription factor (*Cla97C02G033390*), one encoding a GRF transcription factor (*Cla97C02G042620*), one encoding the ABA pathway-related gene *SnRK2-4* (*Cla97C10G186750*) and one encoding *GP-2* (*Cla97C05G105810*) in the starch and sucrose metabolism pathway (**Table 1**).

**Table 1:** Information on the annotations of candidate genes.

Gene id	Gene name	Functional annotation
Cla97C01G021480	UTP6	Maturation of SSU-rRNA from tricistronic rRNA transcript
Cla97C01G005380	LRR-RLK	Protein phosphorylation
Cla97C01G004850	TMKL	Protein phosphorylation
Cla97C05G088130	DUF247	Response to environmental stimuli and biological stress
Cla97C05G105810	GP	Carbohydrate metabolic process
Cla97C10G186750	SnRK2	Plant response to abiotic stresses
Cla97C01G002440	MYB	Plant response to abiotic stresses
Cla97C01G003910	Peptidase C13	Proteolysis
Cla97C01G010410	TK1	Phosphate-containing compound metabolic process
Cla97C10G189700	PABP	Important regulators of readthrough at the premature termination codon
Cla97C03G068160	bHLH	Involved in plant growth and development and response to stress
Cla97C02G033390	HSP	Maintain cell homeostasis
Cla97C02G036610	aminoacylase-1	Cellular amino acid metabolic process
Cla97C02G042620	GRF	Plant growth, development, stress and hormone signaling responses
Cla97C04G069680	Pol II	Transcribe protein-coding mRNA and some noncoding RNA

### qRT−PCR and RNA−seq correlation

**Figure 9:**
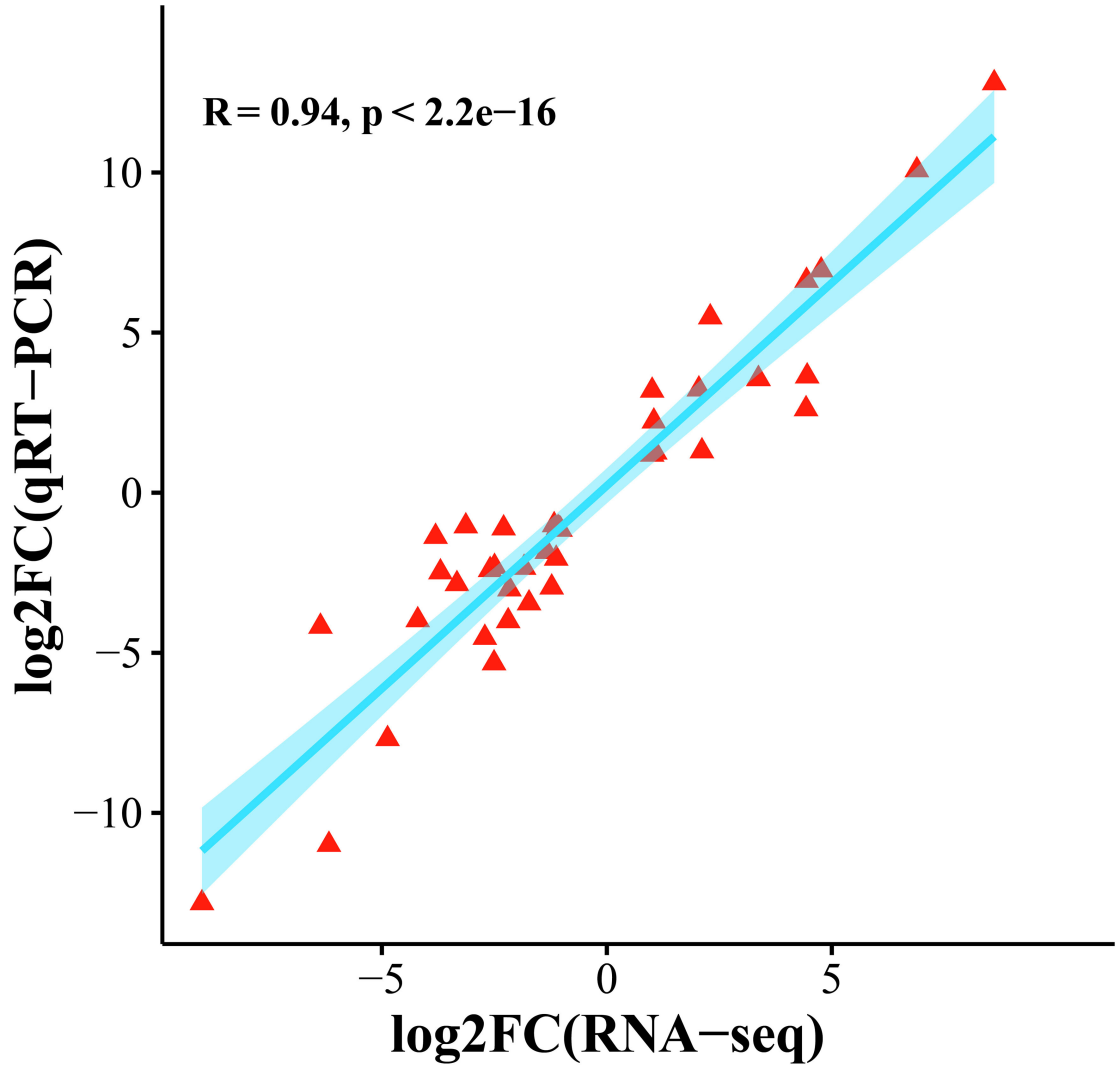
Scatter plot of the correlation between the transcriptome and qRT−PCR gene expression levels.

To confirm the accuracy of the transcriptome expression profile, 10 genes were randomly selected for qRT−PCR analysis. Three independent replicates were performed, and the correlation between the qRT−PCR results and the fold difference in RNA-seq data was calculated. The results showed that the transcriptome data and qRT−PCR data were significantly correlated (R=0.94, p < 0.01), and these comprehensive results indicated that the transcriptome sequencing data were reliable (**Figure 9**).

## Discussion

With global warming and increasing water scarcity, drought stress can severely affect the yield and quality of watermelon plants and has become one of the main problems in watermelon cultivation (Malambane et al., 2023). With the development of sequencing technology and molecular biology, analyzing the molecular mechanisms of watermelon drought resistance and identifying functional genes are important for overcoming this problem (Song et al., 2015). The morphological characteristics of plants are comprehensive reflections of their external responses to environmental changes. Several studies have shown that with the intensification of drought stress, the leaf area and chlorophyll content in the leaves of plants significantly decrease, which leads to a decrease in the rate of photosynthe sis, thereby reducing organic matter accumulation and slowing plant growth (He et al., 2021a; Hu et al., 2022). Under drought stress, improving the efficiency of water use is an effective measure for improving plant drought resistance (Meng et al., 2019). Plants can improve drought resistance by increasing water uptake or reducing water loss, and stomata play an important role in this process. Plants can regulate the efficiency of water use by regulating the opening and closing of stomata and stomatal density, thereby regulating plant drought resistance (Lawson and Vialet-Chabrand, 2019). The decreases in Pn, Tr, Ci, and Gs indicated that drought suppressed the photosynthetic potential of watermelon plants, which may be directly related to the closure of stomata due to drought stress. Some studies have shown that there is a significant positive correlation between watermelon yield and photosynthetically active radiation, with yield reductions ranging from 17.46% to 38.08%. Previous research has shown that the photosynthetic CO_2_ fixation rate decreases by 120% after 24 hours of natural drought stress. After watering for 3 days after drought stress, photosynthesis recovered very slowly, reaching only 50% of the original rate (Mo et al., 2016). This study revealed that after 24 h of PEG stress, the net photosynthetic rate was only 30% of that before stress. This change may be a result of the stress intensity of PEG-simulated drought stress, which was greater than that of natural drought stress. Some studies have also shown that the pattern of change in the Pn under drought stress conditions is consistent between drought-resistant and drought-sensitive plants. However, after rewatering, the Pn of drought-resistant plants recovered to 45.36% of that of control plants, while the Pn of drought-sensitive plants recovered to only 12.71% (Li et al., 2019). The Gs and Tr decreased faster in the first 2 days for drought-resistant materials than for drought-sensitive materials (Li et al., 2019). The water content of watermelon leaves did not decrease under drought stress for 3 days. This result indicates that some solutes may accumulate to reduce the water potential, but the accumulation is not significant because the turgor pressure decreases with decreasing leaf water content. Eight days after the beginning of stress, the water content of the fourth leaf remained at 81% (Kawasaki et al., 2000). Taken together, our results show that in the early stage of drought (0~24 h), due to the severe lack of water in the watermelon body, the stomata on the leaf surface close, which affects the absorption of carbon dioxide and transpiration and mediates drought resistance.

To study the potential connection between the DEGs and metabolites in watermelon under drought stress, transcriptomic and metabolomic analyses were performed. Cluster analysis and PCA revealed that samples from the five time points (0 h, 1 h, 6 h, 12 h and 24 h) before and after drought stress could be divided into 3 groups. Six hours might be the early stage of watermelon response to drought stress, and many DEGs and DRMs were detected at this stage. An independent study was performed on samples of rice and *Lolium multiflorum* that were subjected to drought stress for 6 h (Liu et al., 2022; Dwivedi et al., 2023). Drought stress for 6 h constitutes the early stage of drought withdrawal and may be an important period during which plants respond to drought stress. K-means clustering of the DEGs revealed 1457 DEGs that were specifically expressed at 6 h. Among these DEGs were genes involved in phenylalanine metabolism, carbon fixation in photosynthetic organisms, glycolysis and gluconeogenesis, and the MAPK signaling pathway. The phenylalanine metabolism pathway is a secondary metabolism pathway in plants that is important for secondary metabolite synthesis, and its downstream branching pathways are divided into the flavonoid synthesis and lignin synthesis pathways (Han et al., 2022). *CcCIPK14*-*CcCBL* enhances the drought resistance of pigeonpea plants by promoting the expression of the flavonoid biosynthesis gene *VlbZIP30*, which enhances drought resistance by activating the expression of lignin biosynthesis genes and increasing lignin deposition, as well as activating the expression of drought stress genes (Meng et al., 2021). Lignin enhances drought resistance by altering the degree of cell wall lignification. Although the growth of plant roots depends on the transport of photosynthetic products to aboveground parts, sugar metabolism can improve drought resistance by regulating the root-to-shoot ratio (Du et al., 2020). Our data also showed that 6 h is an important period for watermelon drought stress, and genes related to the abovementioned metabolic pathways could be key research targets.

ABA is synthesized mainly in leaf vascular bundle tissues and then transported to guard cells to induce stomatal closure under conditions of water deficiency. Changes in endogenous ABA levels play a key role in the ABA-dependent dehydration stress response (Lim et al., 2015). The biosynthesis and catabolism of ABA have been observed in many plant species. The metabolomic data revealed that the ABA content of watermelon also increased under drought stress. Analysis of the correlations between genes and metabolites and WGCNA revealed that *SnRK2-4* might be a key gene in watermelon based on its ability to regulate ABA-dependent drought resistance. SnRK2 plays an important role in plant drought resistance. Many studies have reported the function of SnRK2 in drought resistance in different plant species (Zhang et al., 2014; Wang et al., 2015; Feng et al., 2019; Shao et al., 2019). Overexpression of *MpSnRK2.10* significantly enhanced the drought resistance of apple plants (Shao et al., 2019). Similarly, the drought resistance of transgenic plants overexpressing *BdSnRK2.9*, *NtSnRK2.1* and *TaSnRK2.9* significantly increased (Zhang et al., 2014; Wang et al., 2015; Feng et al., 2019). In addition, the overexpression of *TaSnRK2.3* in Arabidopsis increased both the water retention ability (WRA) and the chlorophyll and proline contents in plants under drought stress, as did longer tap roots and more lateral roots (Tian et al., 2013). *OsSAPK2* in rice improved ROS scavenging ability by promoting stomatal closure and upregulating the expression of stress response- and antioxidant enzyme-related genes to adapt to drought stress (Lou et al., 2017). The overexpression of *AtSnRK2.8* increased the tolerance of poplar plants to drought stress (Chun et al., 2014). Taken together, these results indicate that SnRK2 plays a critical role in regulating the plant response to drought stress. To this end, in-depth studies on *SnRK2-4* in watermelon should be conducted in the future.

Transcription factors can regulate the expression of many genes related to plant stress resistance and have attracted increased amounts of attention. Many studies have shown that transcription factors such as bHLH, NAC, WRKY, MYB, and HSP play important roles under abiotic stress. Compared with those of wild-type alfalfa, the drought resistance of *MsMYB*-overexpressing plants increased, and the biomass and quality of the plant improved (Shi et al., 2024). Overexpression of *SlbHLH96* in tomato improves drought resistance by stimulating the expression of genes encoding antioxidants, ABA signaling molecules, and stress-related proteins (Liang et al., 2022). Under drought, ABA signaling activates *MdbHLH160*-mediated expression of *MdSOD1* and *MdDREB2A*-like by promoting the stability of the *MdbHLH160* protein, thereby positively regulating apple drought tolerance (Mao et al., 2024). The overexpression of *IbbHLH118* in potatoes reduced plant tolerance to drought stress, while the overexpression of *IbbHLH66* improved plant tolerance to drought stress. Further research revealed that both genes can interact with the ABA receptor *IbPYL8*, thereby activating ABA signal transduction and improving potato tolerance to drought stress (Xue et al., 2022). The *OsHSP50.2* gene enhances rice tolerance to drought stress by regulating ROS homeostasis (Xiang et al., 2018). The *GhHSP70-26* protein promotes the cotton drought stress response by reducing the degree of cell membrane damage and cell damage from ROS stress (Ni et al., 2021). The 15 candidate genes detected included the MYB, bHLH and HSP transcription factors; however, the exact roles of these genes in watermelon drought resistance remain to be determined.

Sucrose accumulation can improve the drought resistance of plants. Under severe drought stress, the sucrose content in plant cells increases rapidly, which protects cells (Chen et al., 2022). At the cellular level, drought signals promote the production of metabolites such as proline and trehalose, stimulate the antioxidant system to maintain redox homeostasis, and prevent cell damage and destruction of membrane integrity through oxidases (Gupta et al., 2020). *In vitro* studies have shown that disaccharides such as sucrose and trehalose can stabilize enzyme activity and protect membrane structure in the dry state; thus, disaccharides are important desiccation protectants in plants. Studies on wheat species have shown that under drought stress, the soluble sugar content and sucrose synthase (SS) activity significantly increase in the endosperm of these two plant species. The sucrose content and total starch content also increase under drought treatment (Li et al., 2023b). Starch and sucrose metabolism play important roles in providing quinoa with drought resistance (Huan et al., 2022). Our joint analysis revealed that the starch and sucrose metabolism pathway is one of the key pathways affecting the drought resistance of watermelon plants. A network of DEGs and DRMs in the starch and sucrose metabolism pathway was constructed using the screening criteria of PCC ≥ 0.75 and P < 0.05. In summary, these findings provide new insights into drought resistance in watermelon and lay the foundation for in-depth analysis of the molecular mechanisms of watermelon drought resistance.

## Conclusion

In this study, RNA-seq and metabolomic analyses of watermelon plants at five time points before and after drought stress were performed. The findings revealed that 6 h was the critical period for watermelon drought resistance and revealed several important regulatory pathways involved in watermelon drought resistance by identifying DEGs and DRMs before and after stress. K-means clustering was performed to divide DEGs into different clusters, and these clusters could be used to distinguish different time points after stress. In addition, through joint RNA-seq and metabolomic analyses combined with WGCNA, 15 candidate genes associated with watermelon drought resistance, including 4 TFs, were identified. However, the exact role of these genes in watermelon drought resistance has yet to be determined. Our results provide a theoretical basis for in-depth identification of the molecular mechanisms involved in watermelon drought resistance and provide new genetic resources for the study of watermelon drought resistance.

## Data availability statement

The RNA-seq data presented in the study are deposited in the NCBI repository, accession number PRJNA1055702.

## Author contributions

SC: Conceptualization, Data curation, Formal Analysis, Methodology, Software, Writing – original draft, Project administration, Resources, Supervision, Validation. KZ: Conceptualization, Data curation, Investigation, Software, Writing – original draft. YL: Conceptualization, Data curation, Investigation, Writing – original draft. CB: Conceptualization, Data curation, Investigation, Writing – original draft. ZX: Data curation, Investigation, Methodology, Project administration, Software, Validation, Writing – review & editing. YW: Conceptualization, Formal Analysis, Funding acquisition, Resources, Validation, Writing – review & editing.

## Supplementary material

Supplementary Figure 1 PCA of RNA-seq data.

Supplementary Figure 2 Histogram of all metabolic classifications.
