# Supplementary figures and images for: Joint transcriptomic and metabolomic analysis provides new insights into drought resistance in watermelon (Citrullus lanatus)
Source: Front Plant Sci. 2024 May 3;15:1364631. doi: 10.3389/fpls.2024.1364631 (PMC11102048; doi:10.3389/fpls.2024.1364631)

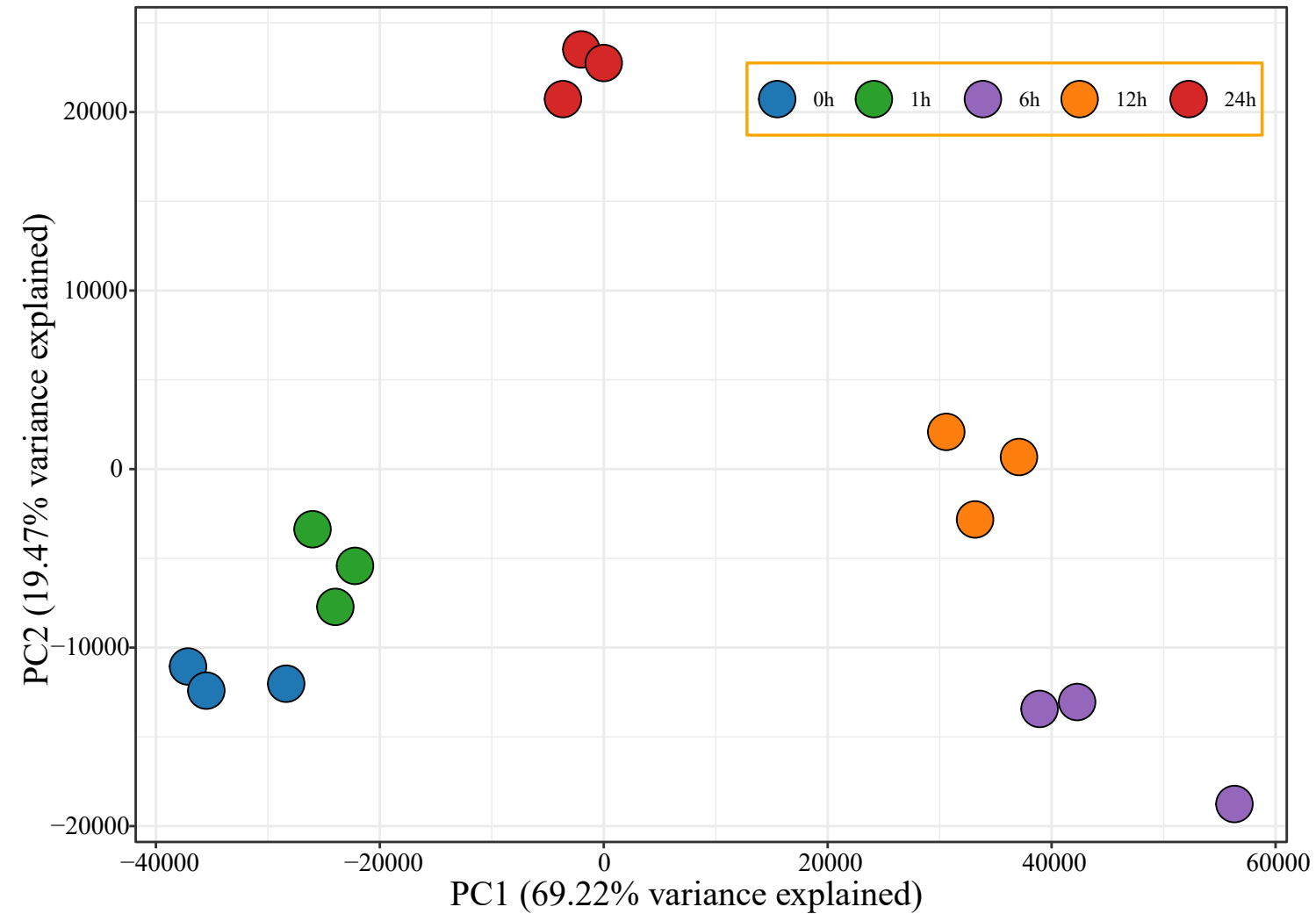

Supplement: Supplementary Figure 1 — PCA of RNA-seq data. [file DataSheet_1.zip › Additional files/Fig S1.pdf]

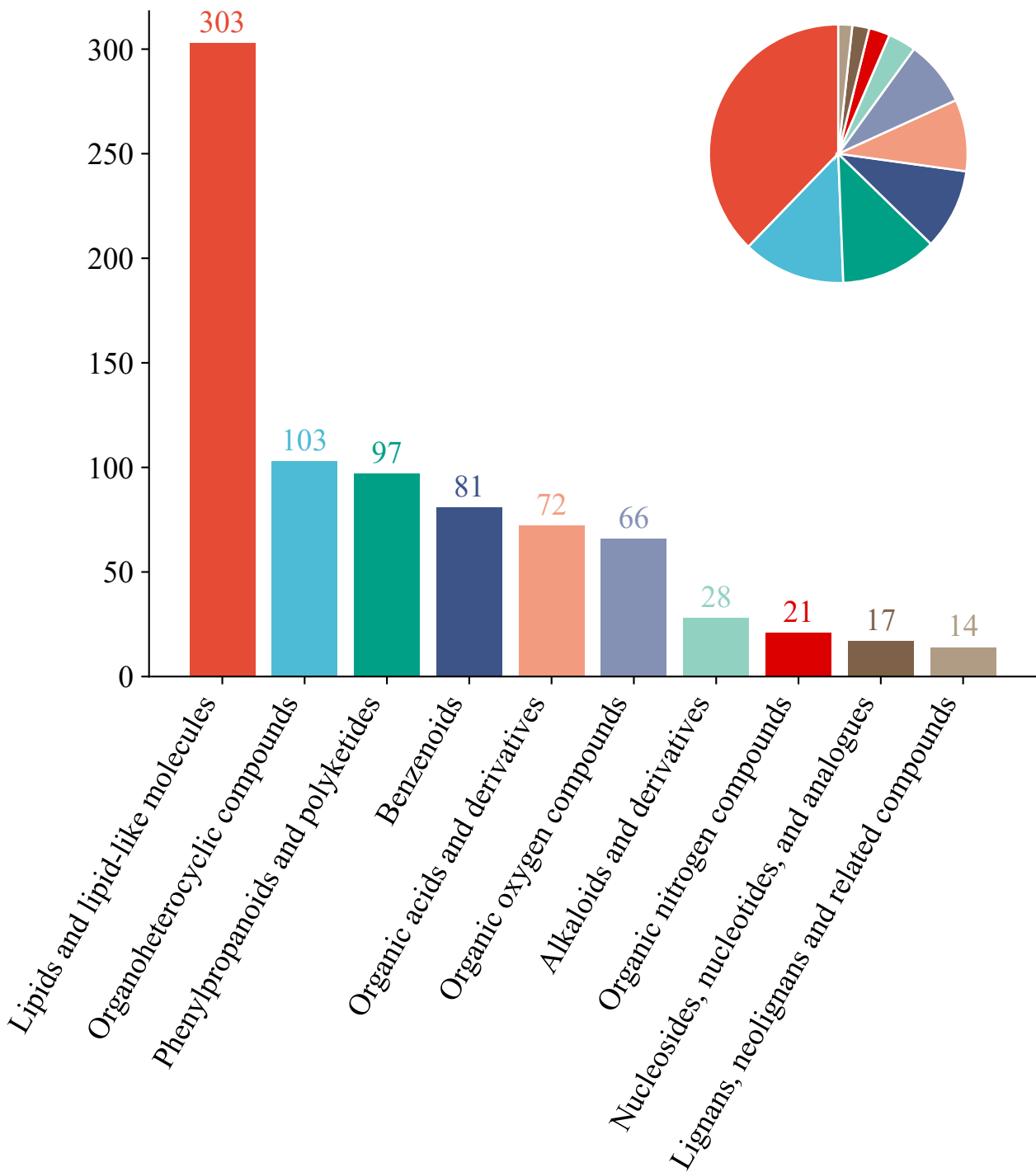

Supplement: Supplementary Figure 1 — PCA of RNA-seq data. [file DataSheet_1.zip › Additional files/Fig S2.pdf]
